# Polyelectrolyte membranes based on phosphorylated-PVA/cellulose acetate for direct methanol fuel cell applications: synthesis, instrumental characterization, and performance testing

**DOI:** 10.1038/s41598-023-40035-6

**Published:** 2023-08-10

**Authors:** Mahmoud Khalaf, Ahmed M. Saeed, Ahmed I. Ali, Elbadawy A. Kamoun, Alaa Fahmy

**Affiliations:** 1 Chemistry Department, Faculty of Science, Al-Azhar University, Cairo, Egypt; 2 Basic Science Department, Faculty of Technology and Education, Helwan University, Saray–El Qoupa, El Sawah Street, Cairo, 11281 Egypt; 3 Polymeric Materials Research Department, Advanced Technology and New Materials Research Institute (ATNMRI), City of Scientific Research and Technological Applications (SRTA-City), New Borg Al-Arab, 21934 Alexandria Egypt; 4 Nanotechnology Research Center (NTRC), The British University in Egypt (BUE), El-Sherouk City, Cairo, 11837 Egypt

**Keywords:** Chemical engineering, Energy

## Abstract

Designing and synthesis of cost-effective and improved methanol permeable and proton conductive membranes are the main challenges for preparation of polymeric electrolyte membrane (PEM). Herein, a cost-effective PEM membrane based on phosphorylated polyvinyl alcohol (PVA)-grafted-cellulose acetate (CA) was prepared by a solution-casting technique. Water and methanol uptakes of phosphorylated PVA/CA membranes were characterized as function with the molar ratio of CA. Additionally, structure and morphology of phosphorylated PVA/CA (Ph-PVA/CA) membranes were verified by FT-IR analysis, SEM investigation. Furthermore, ion exchange capacity (IEC), proton conductivity and methanol permeation of Ph-PVA/CA membranes were examined based on the concentration of OPA basically. The results manifested a perceptible improvement in proton conductivity from 0.035 to 0.05 S/cm at 25 and 70 °C, respectively using 600 μL of OPA, and IEC of 2.1 meq/g using 400 μL of OPA at ambient temperature. On the other hand, methanol permeability (P = 1.08 × 10^–10^ cm^2^/s) was lower than Nafion 117 admirably. The optimum OPA concentration was 200 μL according to conductivity measurements (at 10% PVA, 150 μL GA, and CA 7%). Finally, prepared Ph-PVA/CA membranes exhibited enhancement in critical natures such as proton conductivity and IEC combined with its low-cost materials, which make them excellent candidate as PEM for DMFCs application.

## Introduction

The result of the struggle for energy was intense efforts towards finding a new source. One of the primary energy sources is a fuel cell; which is regarded as a promising candidate for renewable energy generation systems; in addition to having high and distinctive conversion energy from the fuel^1–3^. Fuel cells have various types; however, they can be classified depending on the type of used electrolyte, system requirements, mode of operation and performance. Each type of fuel cells operates on different temperature range, powered by different fuels, requires different kinds of catalyst and therefore, undergoes quite distinguished electro-chemical reactions^4^. The most common types of fuel cells include: (1) solid oxide fuel cell (SOFC), (2) molten carbonate fuel cell (MCFC), (3) phosphoric acid fuel cell (PAFC), (4) alkaline fuel cell (AFC), (5) polymer electrolyte membrane fuel cell (PEMFC), and (6) direct methanol fuel cell (DMFC)^5–8^.

The backbone of DMFC is a proton conducting membrane called polymer electrolyte membrane (PEM) which serves as a thin and solid electrolyte barrier for strict permeation of protons. Therefore, the primary role of the PEM is to transport hydrogen ions between the anode and cathode and separate methanol and oxidant. A perfect PEM should have high proton conductivity, high water uptake, low permeability to the fuel, excellent mechanical and chemical stability, high durability, and the ability to be fabricated into a membrane electrode assembly (MEA)^9–11^. With all these characteristics, must not overlook the commercial aspect so that the cost is as low as possible. The main challenge point is that preparation of a cheaper alternative membrane with high performance^12,13^. The most used polymer membrane for DMFC applications during the past few years was the perfluoro sulfonated polymer electrolyte is Nafion based membranes. Nafion membranes have high chemical stability, good mechanical and thermal stability and good proton conductivity under fully hydrated conditions. They also have a high fuel permeation leading to reduced DMFCs performance in addition to its high cost^14–19^. To overcome these obstacles, the researchers tried to find suitable alternative materials to Nafion through the following set of strategies: (i) blending of Nafion with an appropriate polymer or preparation Nafion-based composites and nanocomposites^20,21^, (ii) applying a barrier layer onto one or both surfaces of the membrane^22,23^, (iii) using an alternative polymer electrolyte^24,25^. Among all these strategies, using polyvinyl alcohol (PVA) as a promising candidate was an inventive choice. PVA membranes have an excellent film, relatively low cost, wide-ranging crystallinity, good oxidative and hydrolytic stability. They also displayed better methanol permeability properties than Nafion^26–28^. While PVA itself has two problems; there are no fixed negative charges like sulfonic acid (–SO_3_H) and carboxylic acid (–COOH) groups and super hydrophilic. So, it swells quickly in water, which makes the proton conductivity and mechanical properties of pure PVA membrane needs an improvement but without destroying its other properties at the same time^29,30^. For this reason, PVA-based membranes for PEM applications can be modified through the incorporation of proton sources to enhance their proton conductivity and swelling properties by blending, crosslinking, or preparing organic–inorganic hybrid membranes^31,32^. For example, PVA-based membrane doped and cross-linked with phosphotungstic acid (HPW) and diethylenetriaminepentaacetic acid (DTPA), as well as PVA-membranes containing hypo phosphorous acid (H_3_PO_2_) as an additive. The properties of these membranes including proton conductivity, ion exchange capacity and swelling properties were improved^33,34^. The general desire to replace the available synthetic polymers with natural biopolymers which are derived from natural raw materials like, plants^32^. Cellulosic materials are highly recommended owing to their abundance in the environment with a predestined total production of 1101–1012 ton/year. Cellulose is a semi crystalline polymer containing crystalline and amorphous phases within the micro fibrils. Hydrogen bonding makes it robust and disable to dissolve in water and most solvents. It even resist to acid and base-catalyzed hydrolysis^33^. In PEM application, cellulosic materials are worthy for their excellent thermal and high glass transition temperature, excellent fuel permeability properties in addition to their ability to retain water for a long time^34^. The researchers discussed cellulose-based electrolyte membranes utilized a lot of cellulosic forms such as bacterial cellulose, cellulose acetate, cellulose nano crystal and cellulose nano fiber. All of these publications have shown and verified that the cellulosic materials are very promising for use in fuel cell applications^35–40^. Cellulose acetate as a base electrolyte is favored to the interaction between hydrogen ion and carboxyl group of cellulose. Moreover, the role of carboxyl group (consisting of carbon, hydrogen, and oxygen) as proton donor and acceptor. In addition, pores, which allows ion transfer and found to have good biocompatibility after blending with other polymers^38^. Despite all these advantages, cellulose has a major drawback wherefore numbers of modifications have been made to improve the ionic conductivity including polymer blending, incorporation of inorganic fillers, cross-linking, sulfonation, phosphorylation and quaternization^39^. Based on the foregoing, this work aims to prepare phosphorylated PVA/CA composite PEMs to prepare a new membrane by mixing two distinct polymers to obtain composite membranes with improved their specific properties. Herein, the available feasibility of phosphorylated PVA/CA composite membranes obtained through chemical crosslinking by glutaraldehyde (GA) were discussed. The effect of OPA as a modifier agent and GA as a crosslinking agent on the resultant membrane properties were studied to find the optimum conditions for PEMs preparation and performance. Furthermore, physiochemical properties of the prepared membranes such as, water and methanol uptake, effect of OPA as a modifier agent, GA as a crosslinking agent, tensile strength, ions exchange capacity (IEC), proton conductivity, and methanol permeability were investigated and discussed well.

## Materials and methods

### Materials

Cellulose acetate (degree of acetylation 40%) and glutaraldehyde solution (grade 1, 70% in water) supplied from Sigma-Aldrich Chemie Gmbh, USA. Orthophosphoric acid, (OPA) (85% purity) was obtained from Poch, Poland. Dimethyl sulfoxide (DMSO) (Assay 99%) delivered by Fisher Scientific, USA. Polyvinyl alcohol (99% hydrolyzed, average MW: 85,000–124,000 g/mol) was supplied from Alpha Chemika, India. Methanol (purity 99.8%) was brought from Fluka Chemie GmbH, Switzerland. Sodium chloride, sodium hydroxide and phenol phthalein are analytical grades were supplied from Arab Solvents Ltd, Egypt.

### Preparation of Ph-PVA/CA PEMs

**Figure 1:**
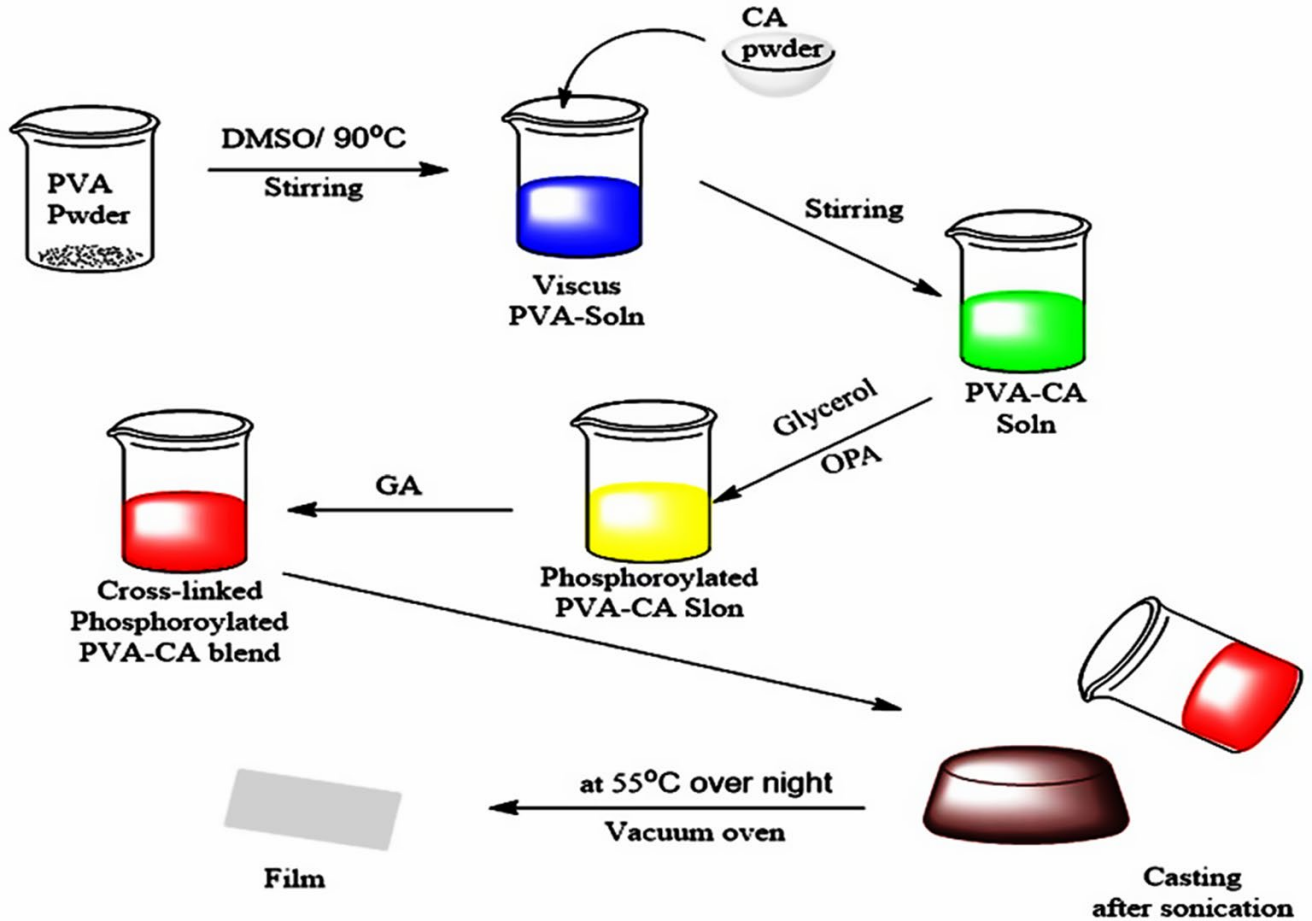
Synthesis procedure of (Ph-PVA/CA) composite PEMs.

Phosphorylated PVA/CA (Ph-PVA/CA) composite membranes were prepared by solution-casting method as depicted in Fig. 1^40^. Typically, 30 g of PVA powder is dissolved in 200 mL of DMSO with continuous stirring at 90 °C for 2 h to get a homogenous solution, and then the PVA solution is increased to 300 mL by DMSO to prepare 10% polymer solution which is then kept under stirring at room temperature. CA solution was prepared by dispersing varying proportions of CA in DMSO solution. PVA/CA blend membrane forming solutions were prepared by mixing their pre-prepared solutions with stirring at room temperature overnight to obtain PVA/CA blends at different ratios of CA (3, 5, 7, and 10 wt/v, %) in the presence of constant concentrations of PVA10 (wt/v,%). Glycerol (0.5 mL) is added as a plasticizer to promote the flexibility and reduce brittleness of blend membranes, followed by reacting with different concentrations of OPA acid for blend modification.

**Figure 2:**
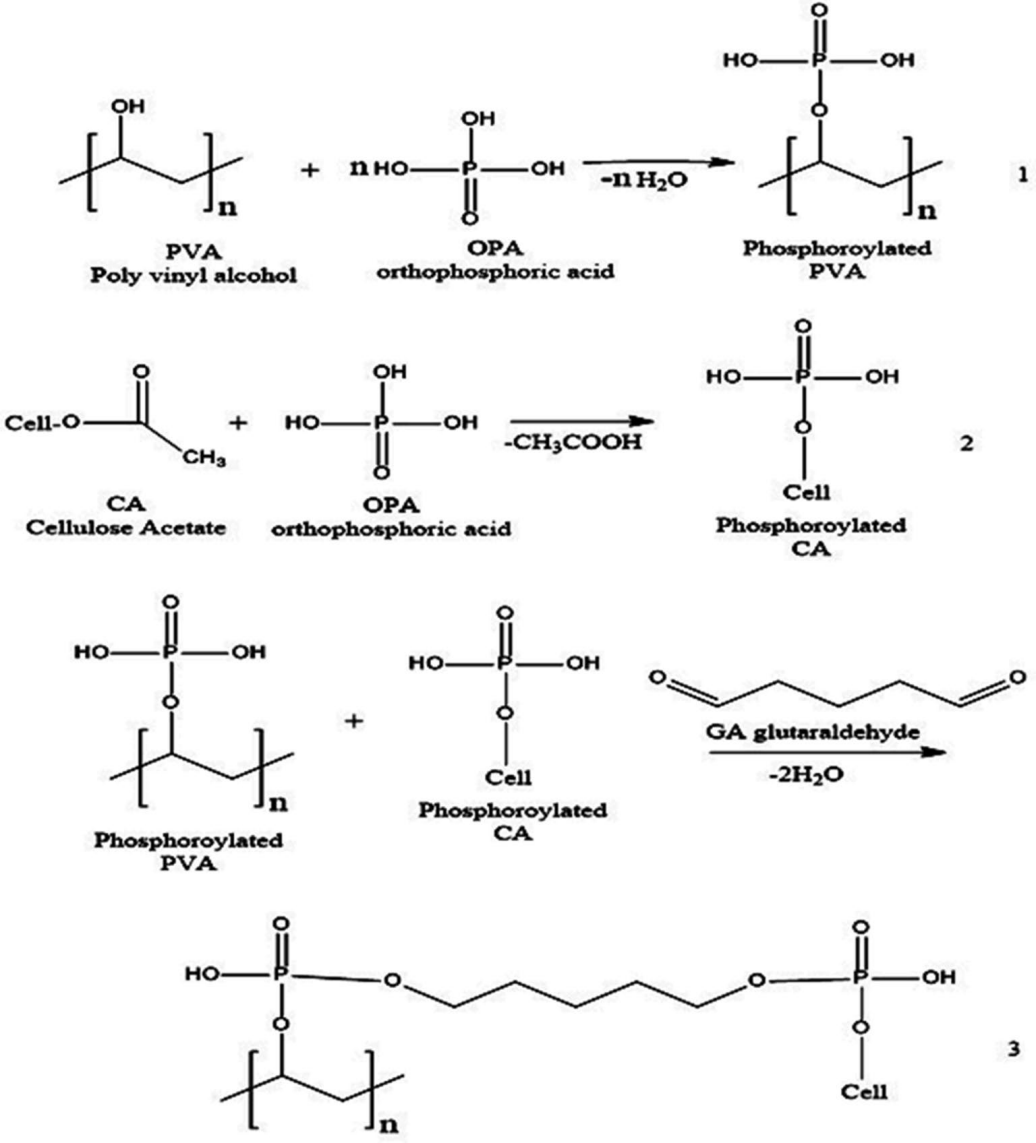
Reaction scheme for preparation of (1) Ph-PVA, (2) Ph-CA, and (3) (Ph-PVA/CA) blend composite membranes.

OPA modifier agent is added to solution stepwise (during a half hour) with continuous stirring at 40 °C for an hour to obtain (Ph-PVA/CA) solution, which is sonicated for 15 min to ensure the homogeneity among compositions and to remove formed air bubbles. GA with different concentrations (50, 100, 150 and 200 μL) are added for crosslinking PVA/CA membranes. The bulk solution is kept under stirring for 1 h till the mixture becomes homogenous and viscous at room temperature, then sonicated again^41^. 15 mL of the phosphorylated PVA/CA blend solutions were poured gently into glass Petri-dishes (diameter of 14 cm) are vacuum-dried at 55 °C for 24 h in a vacuum oven. The resultant phosphorylated PVA/CA composite membranes are gently washed twice with distilled water for further purification and removing the excess of OPA and GA. The dried membranes were kept in plastic bags till use. Figure 2 displays the preparation reactions of Ph-PVA, Ph-CA and (Ph-PVA/CA) blend membranes.

### Membrane characterization

#### FT-IR analysis

The chemical structure analysis of dried membranes and its components PVA/CA were conducted by an FT-IR spectrophotometer (model: Nicolet 6700, Thermo Electron Corporation, Waltham, MA) at a consistent spectral resolution of 6 cm^−1^ over a range of 4000–400 cm^−1^ by taking 116 scans.

#### SEM investigation

The top surface morphologies and microstructures of all Ph-PVA/CA composite polymer membranes were investigated by SEM (type: JEOL, JSM-6360LA, Japan) with 5 kV. The membranes were first coated with Au using an ion sputter coater in (model: 11430, USA, combined with vacuum base unit or SPi module control, model: 11425, USA).

### Physicochemical characterization

#### Water and methanol uptake measurement

(Ph- PVA/CA) films were cut into 2 cm^2^ samples and dried at 40 °C for 12h and then weighed. After this step, these samples are immersed in sealed glass containers filled with deionized water or methanol at ambient temperature for a day. Meanwhile, the samples weighed at different times after removing the sticky drops on their surfaces using tissue paper. Water/methanol uptakes were represented by the divergence in weights before and after immersion and calculated as follow^42^:

1 $$\mathrm{Uptake }\,(\mathrm{\%}) = \frac{\mathrm{Ww}-\mathrm{ Wd }}{\mathrm{Wd}}\times 100,$$

where, W_d_ and W_w_ are the dry and wet weights of tested membrane samples, respectively.

#### Ion exchange capacity (IEC)

Using acid-base titration, measuring IEC was accomplished by soaking an identified weight of the membrane in 20 mL NaCl solution (0.1 M) for 24h to permit the exchange of ions (protons and Na^+^) followed by titration against NaOH_(aq)_ of known concentration, IEC was calculated using following formula^43^:

2 $$\mathrm{IEC }\,(\mathrm{meq}/\mathrm{g}) = \frac{ V \times C }{W},$$

where, *V, C*, and *W* represent the volume of NaOH consumed in titration, the concentration of NaOH, and the weight of the dry film, respectively.

#### Proton conductivity measurement

The proton conductivity of (Ph-PVA/CA) composite membranes was measured using an AC impedance technique with an AC impedance analyzer. The sample membranes were first immersed in 2M H_2_SO_4_ solution at room temperature prior to measurement^42^. Afterwards, films were ensured between two ion blocking platinum electrodes. The surface of the sample was well metallized by a thin layer of Carbone paste to obtain two parallel plates; the sample had the same radius as the electrodes, which are 12 mm in a circular shape, and the thickness is 0.8–1.2 mm. The real (ε′) and imaginary (ε′′) parts of the permittivity of this samples are measured in the frequency range of 100 kHz to 10 Hz with fluctuating voltage of 5 mV at a temperature range from 25 to 80 °C under dry H_2_ and O_2_ atmospheres. Each PVA/CA sample was monitored at least three times. The values of ionic conductivity were calculated by using following equation^44^.

3 $$\upsigma =\frac{\mathrm{ L }}{\mathrm{ R A}},$$

where, σ: proton conductivity (S/cm), A: a cross-sectional area (cm^2^) of the membrane, L: membrane thickness (cm), and R: measured resistance for the samples, respectively.

#### Methanol permeability measurement

**Figure 3:**
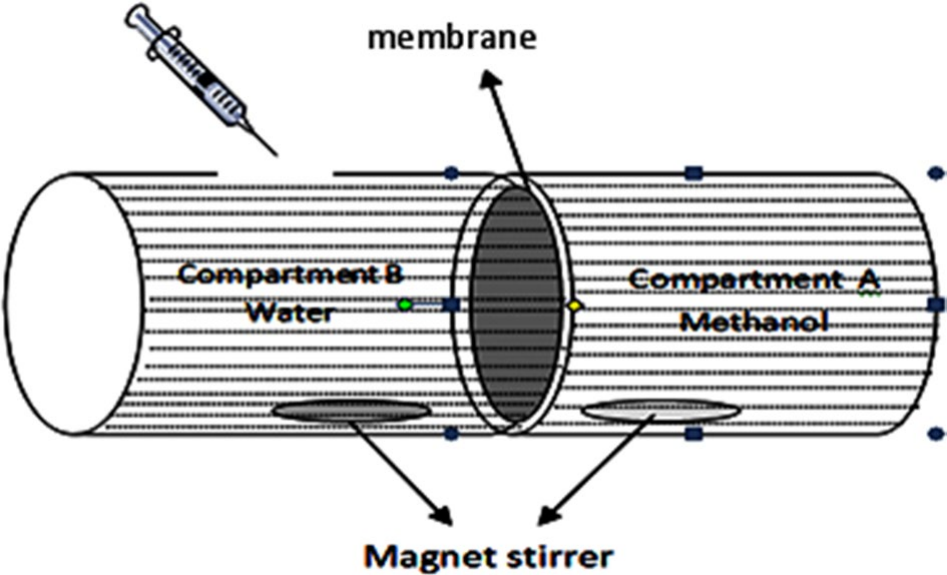
Schematic diagram showing a diffusion cell for methanol crossover measurements.

Methanol permeability measurements simulate what occurs in fuel cells. It refers to the resistance of the membrane to methanol and its ability to prevent or reduce its crossover. Permeation observations were performed using a glass cell with two identical compartments to work as a diffusion cell (Fig. 3). The prepared membranes were placed between the two half-cells with tight closure; each membrane was steeped in a 2 M methanol solution for at least 24 h. The two compartments are namely A and B, compartment A was filled with 2 M Methanol aqueous solution and compartment B was filled with deionized water. The whole content of compartment B was taken away at regular time periods (20 min) and replaced with deionized water. Gas chromatography (GC-PAC, oxytracer Chromatographic Instruments Co., Ltd) was used to determine the methanol concentration of the samples taken from compartment B. The methanol concentration of each sample could be determined using a suitable calibration curve from the area under peaks. Methanol permeability was then calculated from the following equations^45^:

4 $$\mathrm{CB }\,\left(\mathrm{t}\right)=\frac{\mathrm{ A }\times \mathrm{ P }}{ {\mathrm{V}}_{B} \times \mathrm{ L }}{\mathrm{C}}_{A}\left(\mathrm{t}-{\mathrm{t}}_{\text{o}}\right),$$

5 $$\mathrm{P }=\mathrm{ \alpha }\frac{{\mathrm{V}}_{B}}{\mathrm{A}}\times \frac{\mathrm{L}}{{\mathrm{C}}_{A}},$$

where, P: methanol permeability (cm^2^/s), A: cross-sectional area (cm^2^) of the membrane, L: membrane thickness (cm), V_B_: the volume of deionized water (cm^3^), C_A_ & C_B_: the initial concentrations of methanol in the compartments A and B, respectively, t: the time lag, t_o_: the initial time, and α: the slope of the linear function of C_B_ versus t.

#### Thermal gravimetric analysis (TGA)

The thermal characteristics of the prepared membranes have been accomplished employing thermo-gravimetric analysis (TGA). A Shimadzu TGA-50 was utilized to perform the analysis in the temperature range of 35 to 800 °C with a heating rate of 10 °C/min.

## Results and discussion

### Membranes characterization

#### FTIR analysis

**Figure 4:**
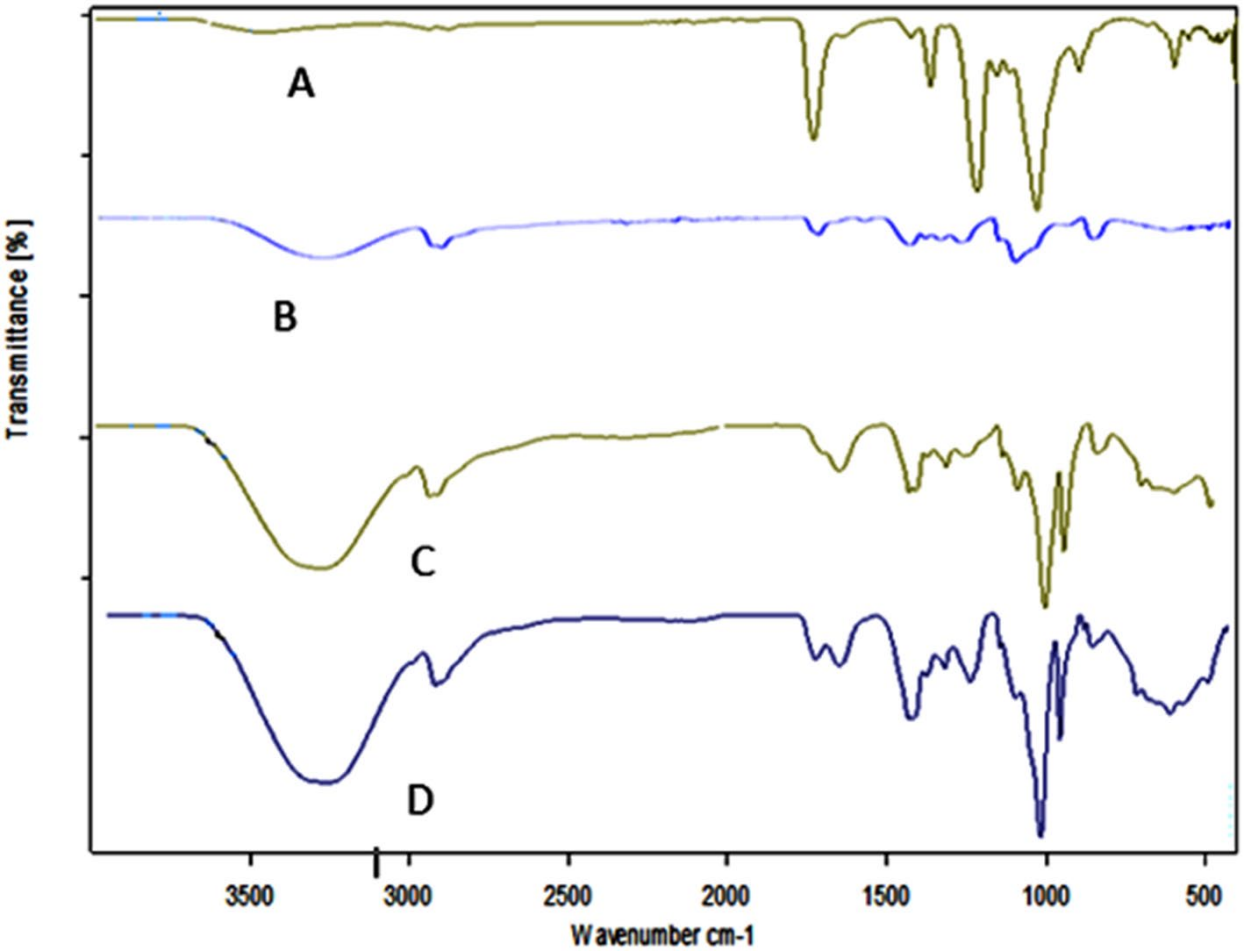
FT-IR spectra of (**A**) pure CA, (**B**) pure PVA, (**C**) Ph-PVA/CA crosslinked membrane and (**D**) non-phosphorylated crosslinked PVA/CA membrane.

FT-IR spectra of PVA, CA, PVA/CA, and crosslinked Ph-PVA/CA membranes were represented in Fig. 4. The spectrum of CA (Fig. 4A) illustrates that a recognized band of O–H group at ν 3455 cm^−1^^46^. The bands have been observed at the wavenumbers ν 1735, 1220, and 1033 cm^−1^ are corresponded to C=O, C–O and C–O–C, respectively. The vibrational band which is observed at ν 2955 cm^−1^ refers to the stretching of CH_2_ and a small sharp band at ν 1368 cm^−1^ indicates the presence of terminal CH_3_ groups^47^. The spectrum of PVA (Fig. 4B) shows that a broad band which is observed at ν 3291 cm^−1^ is related to the stretching of O–H from the intramolecular hydrogen bonds. The characteristic –OH peak of CA was found at around ~ 3455 cm^−1^ wavenumber, where the weak intensity of this peak is due to the degree of acetylation of CA which reduces the amount of OH groups^48^. The vibrational band predestined at ν 2909 cm^−1^ refers to the stretching of C–H in alkyl groups and at ν 1420 cm^−1^ indicates the presence of CH_2_ in the backbone of polymer chains. The peak at ν 1712 cm^−1^ is due to stretching of C=O groups, in addition to presence a band at ν 1087 cm^−1^ which might be referred to the C–O–C^49^. The spectra of both PVA/CA (Fig. 4D) and Ph-PVA/CA (Fig. 4C) crosslinked by GA are scanned by FT-IR to observe the changes which occur in the structure. The OH peak of pure CA was shifted in PVA/CA blend indicating interaction between the two polymers^46^. It was observed that the intensity of OH group in crosslinked Ph-PVA/CA membrane was lowered in non-Phosphorylated PVA/CA membrane indicates the exhaustion of –OH group in functionalization step by OPA through formation of covalent bond with –OH groups^50,51^. Furthermore, a weak band was observed at ν 1654 cm^−1^ which signifies to P=O in OPA and C=O in GA which in turn improves the movement of electrons^45^. While the absorption band at ν 1000 cm^−1^ indicates the presence of C–O–C with high intensity which proves consumption of –OH groups.

#### SEM investigation

**Figure 5:**
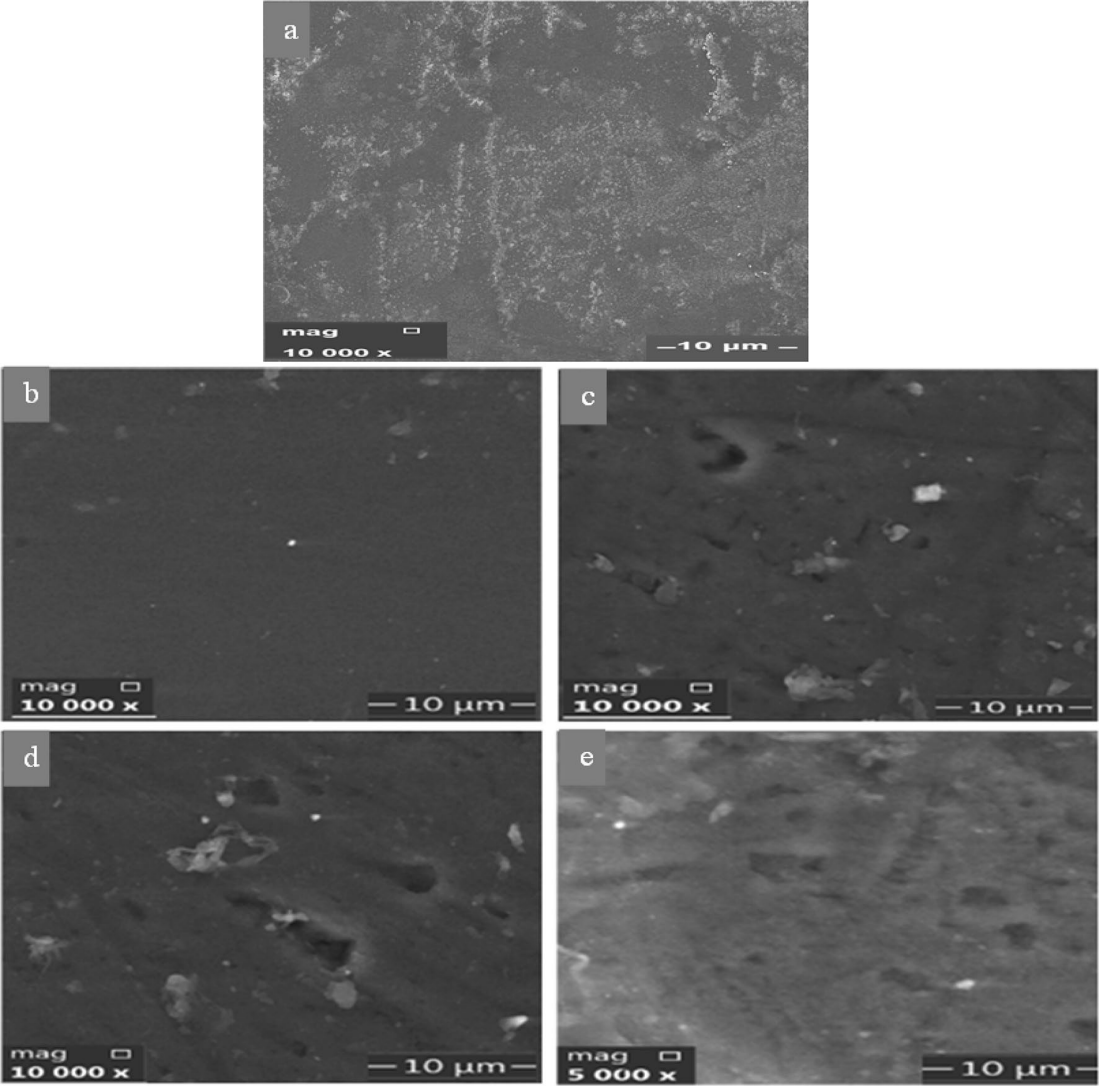
SEM images of pure PVA (**a**), the membranes PVA/CA (**b**), crosslinked Ph-PVA/CA membranes using 200 (**c**), 400 (**d**), and 600 μL of OPA (**e**) as modifier phosphorizing agent.

Figure 5 displays SEM images of pure PVA (Fig. 5a), PVA/CA (Fig. 5b) and crosslinked membranes with different OPA concentrations (200, 400 and 600 μL) (Fig. 5c–e); respectively. The surface morphology with a smooth appearance of PVA/CA membrane indicates that the polymer matrix components are homogeneous and compatible (Fig. 5b). In contrast, with adding OPA to the matrix, the morphology of membranes surface changed to become a somewhat rougher and irregular shape structure (Fig. 5c–e), which proves the changes in the PVA/CA membranes due to the phosphorylation process as previously reported by Rosli et al.^52^. White dots that appear in the images might be related to the dust from air as found in SEM images of membranes in some literatures as in references^53,54^.

### Physicochemical properties of PEMs

#### Water and methanol uptake

**Figure 6:**
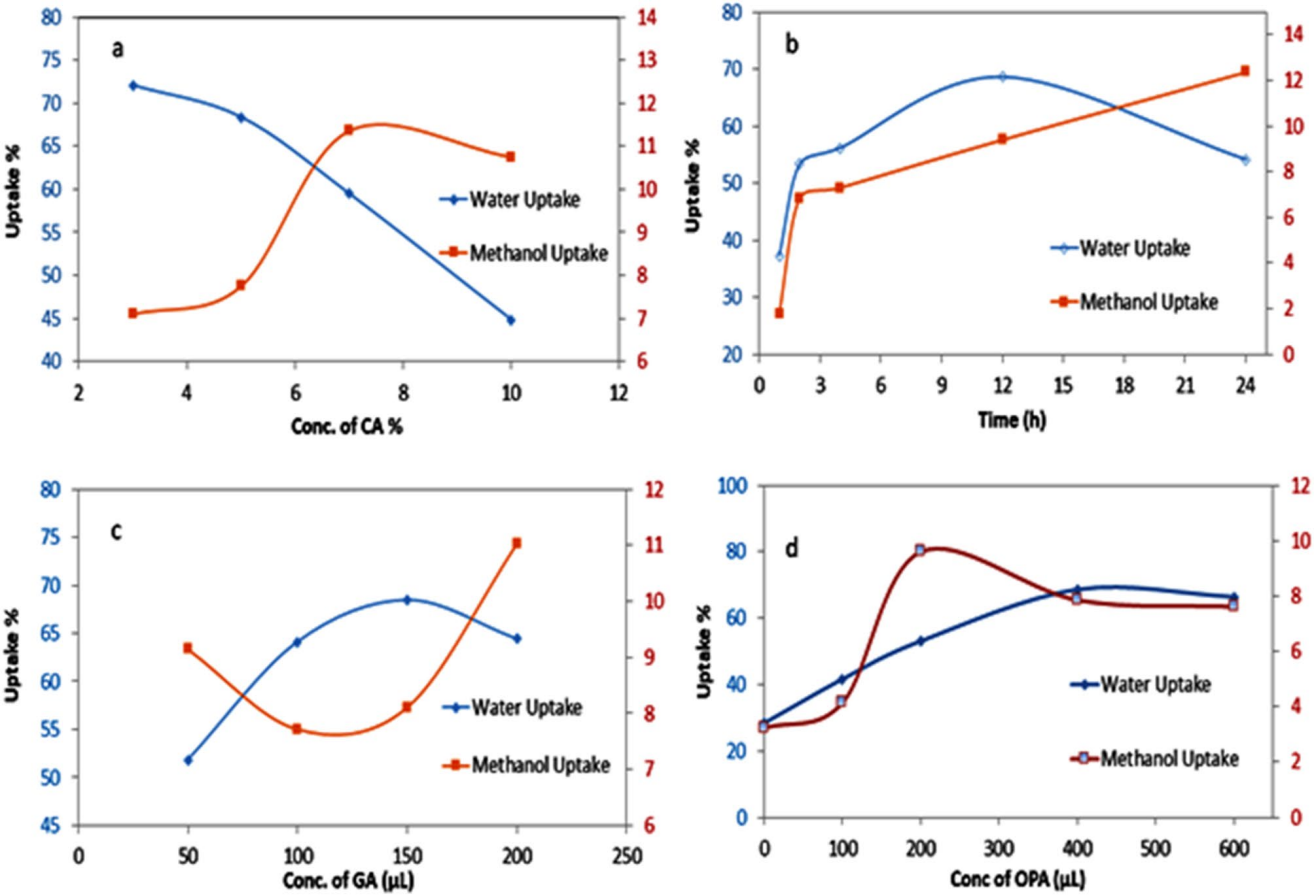
Water and methanol uptake with **(a)** different concentration of CA (3, 5, 7 and 10 wt/v, %) where membranes were prepared with the following conditions (10% PVA, 100 μL GA, 450 μL phosphoric acid, for 24 h), **(b)** with different times (1, 2, 4, 12, and 24 h) where membranes were prepared as (10% PVA, 100 μL GA, 5% CA, and 450 mL phosphoric acid), **(c)** with different GA concentrations (50, 100, 150, and 200 mL) where membranes were prepared as (10%PVA, 5% CA, 450 M OPA after 12 h) and **(d)** various concentrations of OPA.

Water/methanol uptake (WU% and MU%) capacity is one of the most critical parameters for the ionic transport of PEM and fuel crossover in DMFCs^55^. Water and methanol uptake of Ph-PVA/CA membranes as a function of CA ratio was illustrated in Fig. 6a. The results show that with increasing the amount of CA, the water uptake values decrease due to the crosslinking of PVA with GA and phosphoric acid^51^; in addition to the presence of CA which narrowing the transport channels. However, the methanol uptake values of such samples increase up to 7% of CA. It is well known that limited water uptake increases the mobility of H^+^ and accordingly improves proton conductivity due to the ionic nature of water molecules.

Figure 6b shows water (WU) and methanol uptake (MU)% at different times. The results indicate that WU% and MU% increase with time up to 12 h, then WU% reduces and MU% increases after 24 h. This observation is related to the exclusion of water from the membrane at higher methanol uptake^56^. Water uptake of pure PVA after only 30 min reached 460% and after that it was difficult to measure the uptake.

#### Effect of GA as a crosslinker on WU% and MU% of membranes

The increase of GA amount leads to a significant increase of the W/M uptakes of the membranes with fixed ratios (Fig. 6c). It was noticed that the water uptake increases with increasing of GA from 50 to 150 μL, where the best and optimized methanol uptake values are observed in the range from 100 to 150 μL of GA.

#### Effect of OPA on WU% and MU% of membranes

As the amount of OPA increased in PEMs, the water uptake of the membranes increases owing to the hydrophilic nature of OPA^57^. However, it decreases at higher concentrations of OPA than 400 μL (Fig. 6d). The reason for such behavior might be as a result of the compactness of the structure, which in turn avoids water overload in the polymeric matrix channels^51,55^ in addition to the crosslinking feature^52,54,58,59^. On the other hand, MU% was increased up to 200 μL of OPA then it is decreased or almost constant with increasing the amount of OPA due to the hydrogen bonding formation between the methanol molecules and OPA at higher concentrations.

#### Ion exchange capacity (IEC)

The ion exchange capacity (IEC) depends on the number of ion commutable sites distributed throughout the polymer structure and they are responsible for the proton conduction. It is roundabout and effective root to investigate the proton conductivity. The pristine CA and PVA are weak proton conductors because of their functional groups, which are not enough to achieve competitive membranes with high conductivity. The conductivity and IEC of pure PVA were 0.05 S/cm and 0.01 meq/g respectively. So it was resorted to use ortho phosphoric acid to improve this critical property^60^.

##### Effect of OPA concentration on the IEC of PEMs

**Figure 7:**
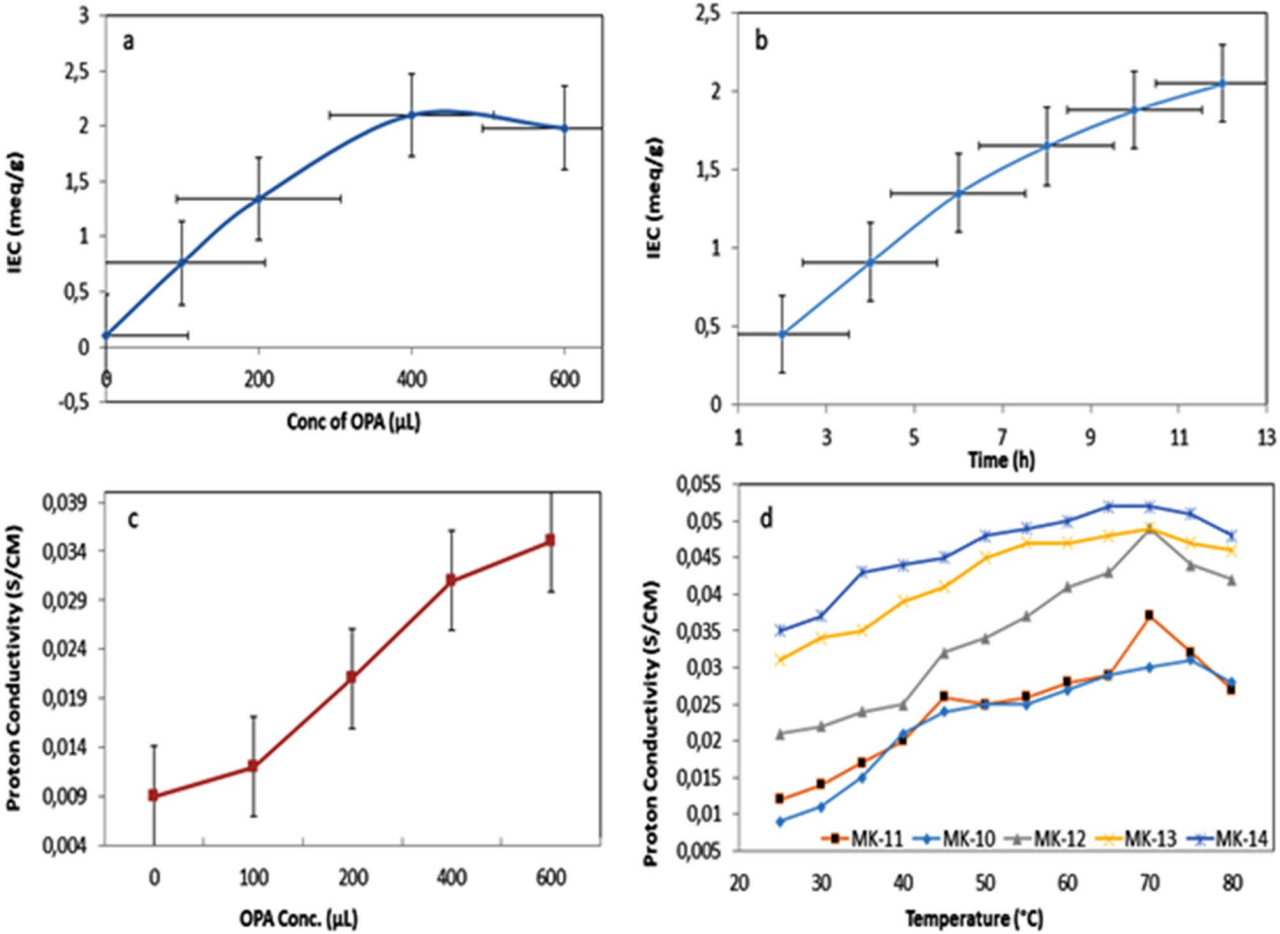
(**a**) Effect of OPA concentration on the values of IEC of Ph-PVA/CA composite membranes, (**b**) effect of reaction time on the IEC of Ph-PVA/CA membrane, (**c**) effect of OPA concentration on the proton conductivity of Ph- PVA/CA composite membranes at ambient temperature, and (**d**) effect ranged temperatures from 25 to 80 °C on the proton conductivity of Ph- PVA/CA membranes as a function of OPA concentration.

Figure 7a reveals that the IEC of Ph-PVA/CA membranes increases distinctly with increasing the amount of OPA as a modifier agent, and the highest IEC is obtained with 400 μL of OPA. Also, increasing OPA concentration creates high ionizing sites between PVA/CA membrane and OPA because the OPA contains phosphate ions PO4^–3^ that leads to increasing the propagation of OPA into the PVA/CA bulk matrix which is responsible for the IEC^57^. Thus, IEC is observed to be decreased beyond ~ 450 μL, the crosslinking might be the reason^57^.

##### Effect of reaction time on the IEC of PEMs

Figure 7b displays the effect of extension of the reaction time on the IEC of Ph-PVA/CA membranes. The augmentation of the IEC values is due to increasing the reaction between hydroxyl and acetyl groups of PVA and CA with OPA molecules leading to exchanging more ions with increased reaction time.

#### Proton conductivity

Proton conductivity is the most important property to evaluate the membrane as PEM. It is proposed that PVA/CA modified with OPA is considered the primary factor to generate the ionic sites which leads to conduct the protons. This might be because the OPA is the donor and the carrier of phosphate groups PO_4_^–3^ (as charged negative ions), which are ultimately responsible for proton conductivity. The membranes have been prepared by varying the OPA concentrations and different temperatures with fixed ratios for other materials in composite membrane (10% PVA, 5% CA, 100 mL GA) and measuring was done by knowing the thickness, the area, and resistance from the impedance data. Here the behavior of the AC conductivities for all samples are similar, where the conductivities are increases linearly with temperature, the conductivity activated by heat, indication to the thermal energy increased the hydrogen ionic conductivities, as a results of moving ions in the samples with temperature. Further, the values of σ are increased with the OPA concentrations as in Fig. 7d^61^.

##### Effect of OPA concentration on the proton conductivity of PEMs

As seen in Fig. 7c, the proton conductivity values range progressively from 0.009 to 0.035 S/cm are owing to the modification of PVA/CA membranes by varied OPA amount from 0 to 600 μL at ambient temperature. In phosphorylated composite membranes, PO_4_^–3^ groups expedite proton transfer principally via the Grotthuss mechanism^55^. It is observed that increasing the concentration of OPA in the membrane leads to an increase in the number of charged negative ions which are responsible for protonic conductivity generation. The contiguous proton in the acid molecule is then attracted to the deficiency and can be conducted to that site. The hydrated or partially hydrated conditions from water uptakes character play a role in the combined vehicle and Grotthuss mechanisms^62^. Notably, additional acid molecules get absorbed during the phosphorylation process as they start to overcrowd in the free volume of the polymeric matrix. These “free phosphoric acids” are also responsible for proton conduction^63^.

##### Effect of reaction time on the proton conductivity of PEMs

Proton conductivities of Ph-PVA/CA membranes, with OPA concentrations of (0, 100, 200, 400 and 600 mL) were assigned as MK10, MK11, MK12, MK13, and MK14; respectively is presented in Fig. 7d, with different temperatures. It is observed that the proton conductivity for all membranes increases at a trend from 25 to 70 °C and then it goes down. The accretion in conductivity at high temperatures is assigned to the mobility chain of polymers which offers more opportunities for proton transfer. The dwindling in the proton conductivity at temperature above 70 °C might be due to the significant water loss from the membrane at high temperatures, and consequently decreasing proton conductivity^64^.

**Figure 8:**
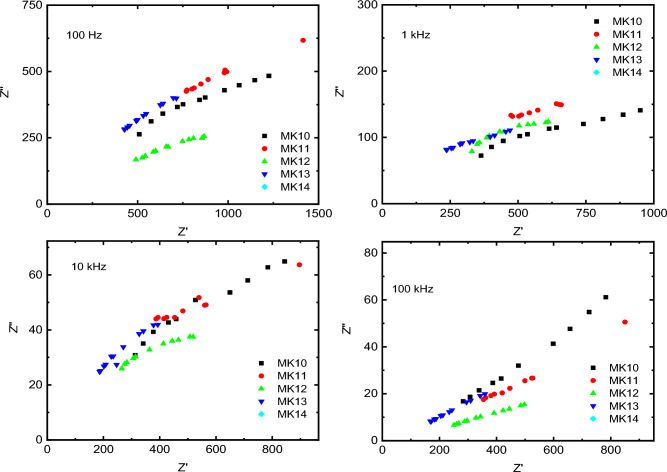
The Cole–Cole plot for the samples of PEMs (with OPA concentrations of (0, 100, 200, 400 and 600 mL) at different frequencies (100 Hz, 1 kHz, 10 kHz, and 100 kHz).

Figure 8 presents the relation between the imaginary part of the impedance (Zʹʹ) and the real part of the impedance (Zʹ) for all samples of PEMs at different frequencies from 100 Hz to 100 kHz. The samples show influence of frequency on the behavior of the impedance, where at lower frequency (100 Hz) the samples exhibited linear behavior, except sample MK12 (with OPA concentrations: 200 μL) presented semicircles (green line). It is known that the linear behavior of Cole–Cole plot indicates a capacitance and resistance connected series with each other, in this case sample MK12 presented a circuit consisting of resistance and capacitor connected parallel. With increasing frequency MK12 has become more ideal to be semicircle. At higher frequency (100 kHz), All samples take linear-shape behavior, this means sample MK12 can be excellent candidate for use in DMFC or PEM at frequency < 100 kHz^65^.

#### Methanol permeability

In addition to obtaining high proton conductivity, the polymeric membrane of fuel cells is also needed to separate the fuel from the oxidant. Perfect DMFC is impermeable to methanol and provides less methanol loss producing higher energy density. Methanol crossover decreases the performance of DMFCs through poisoning the cathode catalyst, fuel efficiency relief, and electrode potential lowering due to oxidation of methanol at the cathode. The permeability of methanol for Ph-PVA/CA membrane with ratio (10% PVA, 5% CA, 100 mL GA, 450 μL OPA) is determined using a diffusion cell. Since the methanol permeable with higher ionic conductivity membranes is the most important characteristic of DMFC. GC analysis was used to calculate the concentration of methanol in the water reservoir to estimate the methanol permeability of the best membrane under investigation^66^.

**Figure 9:**
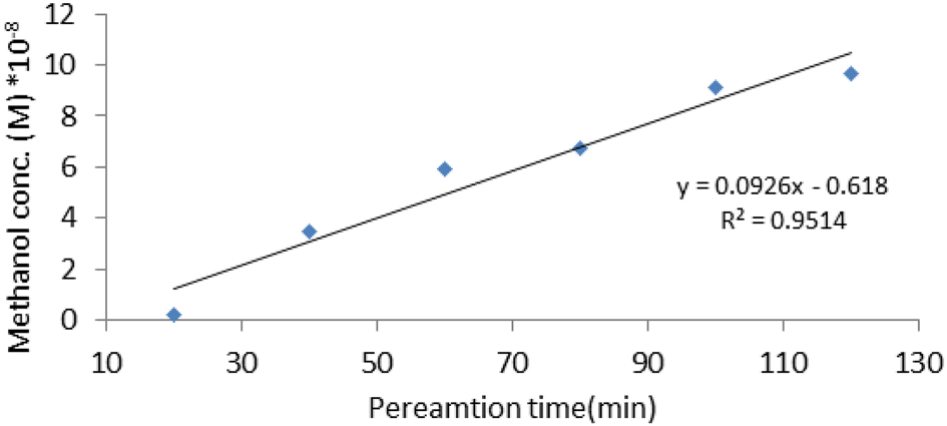
Methanol concentration *vs* permeation time of Ph-PVA/CA membranes, where α = slope = 0.0926, A = 9.62 cm^2^, V_B_ = 100 cm^3^, C_A_ = 8% wt, L = 0.09 cm, and P = 1.08 × 10^–10^ cm^2^/S.

The change in methanol concentration in the water reservoir against the permeation time is represented graphically in Fig. 9 of Ph-PVA/CA membrane. The concentration of methanol in the water compartment is related to the permeation time according to Eq. (6). The slope of the straight line of the change of methanol concentration in the water compartment (CB) against the permeation time (t) assigned as α.

The methanol permeability (P) can be calculated using Eq. (6)^57^; Where CA is the initial concentration of methanol in the compartment (A), A is the membrane working area, L is the membrane thickness and VB is the volume of water compartment.

6 $$\mathrm{P }=\mathrm{ \alpha }\frac{\mathrm{VB}}{\mathrm{A}}\times \frac{\mathrm{L}}{\mathrm{CA}}.$$

Compared to pure PVA (4.12 × 10^–6^ cm^2^/S)^67^, the methanol permeability of Ph-PVA/CA membranes is calculated and was 1.08 × 10^–10^ cm^2^/S indicating that Ph-PVA/CA membranes exhibited lower methanol permeability than Nafion®117 membranes (2.37 × 10^–6^ cm^2^/S)^68^. The decrease in methanol permeability might be closely a result of reducing the vacant space and inducing a much denser structure to act as the methanol barrier, in addition to the crosslinking structure formed between the OPA molecules and the polymer which led to methanol barrier property to be lowered^57^.

#### Thermal stability

**Figure 10:**
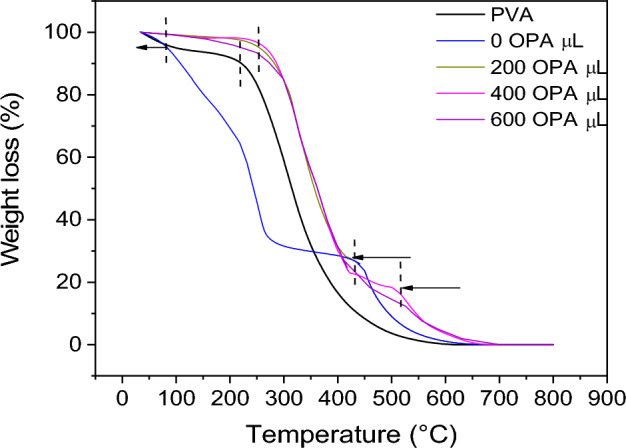
Thermogram of PVA/CA blend polymers membranes in dependance on the OPA value in the membranes.

Thermogravimetric analysis (TGA) was used to investigate the thermal stability of membranes in the wide range of temperature from 35 to 800 °C (Fig. 10). There are two decomposition stages that were detected for pure PVA. The first of them take place in the range from 50 to 130 °C with weight loss of 8% which assigned to the removal of residual water solvents. The last and main stage of decomposition occurs from 250 to 350 °C with weight loss of more than 80% due to the degradation of the chain backbone of the polymer. In the case of PVA/CA blend polymer membrane without OPA (0 OPA), it was observed that the thermal stability of this membrane is reduced and reached 60% weight loss % at 250 °C. It means that the PVA conducted to CA via physical bonds. In addition to the first steps of weight loss at around 100 °C as that was observed for pure PVA. A further stage was detected at nearly 450 °C for PVA/CA blended polymer membranes which might be concerned with the chemical bond between the PVA and CA. A high thermal stability was noticed for the blend polymer based on the presence of OPA values. The most important role of OPA is the cleavage and dehydration of both PVA and CA forming a crosslinked polymer layer.

## Conclusions

Polyvinyl alcohol/cellulose acetate are blended to form a promising composite membrane throughout crosslinking and activation process by using glutaraldehyde (GA) and ortho phosphoric acid (OPA); respectively, which is added to enhance the required features like ion exchange capacity and proton conductivity; respectively. The properties of phosphorylated PVA/CA membrane, especially ion exchange capacity (IEC) and proton conductivity were evaluated in dependence on OPA concentration, time, and temperature, which were monitored and observed to achieve the exemplary conditions. The chemical and morphological structure of the phosphorylated PVA/CA membranes were analyzed using FTIR, and SEM analyses. FT-IR confirmed the interaction between PVA, CA and OPA by changing the site or the intensity of the spectra for all function groups. SEM images displayed changes in the surface morphology due to OPA from smooth to rough surface. IEC of the synthesized membranes acquired in the range 0.7–2.1 meq/g with change OPA concentration from 100 to 600 mL after 12 h. Furthermore, the water and methanol uptake of the phosphorylated PVA/CA membranes were 68.7% and 7.74%, respectively using (10% PVA, 100 mL GA, 5% CA, 450 mL phosphoric acid), for 12 h. The best proton conductivity was achieved at the higher OPA concentration and were around 0.035 S/cm and 0.05 S/cm at 25 and 70 °C, respectively. Finally, the methanol permeation was lower than that of Nafion 117 membranes. The Cole–Cole plot confirming the samples shows linear behavior and sample MK12 is the optimum concentration at frequency < 100 kHz. The obtained results of Ph-PVA/CA membranes make them excellent candidates for use in DMFC or PEM due to the availability and low cost of its components.

## Data Availability

All data generated or analyzed during this study are included in this published article.
